# Current concepts in odontohypophosphatasia form of hypophosphatasia and report of two cases

**DOI:** 10.1186/s12903-016-0266-0

**Published:** 2016-08-17

**Authors:** Zhu-yu Wang, Kai Zhang, Guang-sen Zheng, Wei Qiao, Yu-xiong Su

**Affiliations:** 1 Department of Endodontics, Guanghua School of Stomatology, Sun Yat-sen University, Guangzhou, China; 2 Department of Oral and Maxillofacial Surgery, Guanghua School of Stomatology, Sun Yat-sen University, Guangzhou, China; 3 Division of Oral and Maxillofacial Surgery, Faculty of Dentistry, the University of Hong Kong, 34 Hospital Road, Hong Kong, Hong Kong

**Keywords:** Alkaline phosphatase, Cone-beam computed tomography, Dysplasia, Hyperthyroidism, Radicular cyst, Short root anomaly

## Abstract

**Background:**

Hypophosphatasia is a rare inherited disease derived from mutations in tissue non-specific alkaline phosphatase genes, with typical oral symptoms including short root anomaly and dysplasia of dentin or cementum.

**Case presentation:**

Two young female patients presented with short root anomaly with a history of premature loss of deciduous and/or permanent teeth. The laboratory and imaging investigations were performed. One case was diagnosed as odontohypophosphatasia concurrent with hyperthyroidism, the other was odontohypophosphatasia concurrent with multiple radicular cysts.

**Conclusion:**

This report presents two cases of odontohypophosphatasia, a rare disease which is difficult to be diagnosed, and highlights that the history of premature loss of deciduous and/or permanent teeth, oral manifestation and laboratory tests are crucial for clinical diagnosis.

## Background

Hypophosphatasia (HPP) is a rare inherited disease mainly characterized by bone and tooth dysplasia resulting from low serum level or activity of alkaline phosphatase (ALP), especially tissue non-specific alkaline phosphatase (TNSALP). HPP can occur from perinatal to adult stages and is categorized into 6 forms with different clinical manifestations, including perinatal (lethal), prenatal (benign), infantile, childhood, adult, and odontohypophosphatasia forms [1]. Research has indicated that the incidence of severe forms is about 1/100,000, and the total incidence of the disease is generally underestimated for the reason that mild forms are always ignored [2]. The typical oral symptoms include short root anomaly and dysplasia of dentin or cementum. Some patients may only present with a history of premature loss of deciduous and/or permanent teeth. Therefore a thorough understanding of the disease is of great importance for dentists. In addition to clinical manifestations, biochemical tests and/or the genetic analyses are also crucial to the diagnosis of the disease. We presented two cases of odontohypophosphatasia form of HPP to illustrate the characteristics of this inherited disease. Cone-beam computed tomography (CBCT) was applied for better evaluation of short root anomaly.

## Case presentation

This study was approved by the Institutional Review Board of Guanghua School of Stomatology, Sun Yat-sen University (Reference No. ERC-[2014]-18). Informed consent was obtained from both patients. Dental, medical and family history of two patients with short root anomaly were recorded in detail. Clinical examination and diagnostic radiography were performed at Hospital of Stomatology, Sun Yat-sen University, Guangzhou, China. CBCT was applied to measure the length of the crown and the root of teeth [3]. The laboratory examinations including urinalysis, complete blood count, serum ALP level, serum calcium level, serum calcitonin (CT) level, serum parathyroid hormone (PTH) level, serum free triiodothyronine (FT3) level, serum free thyroxine (FT4) level and serum thyroid-stimulating hormone (TSH) level were conducted.

### Case one

**Fig. 1:**
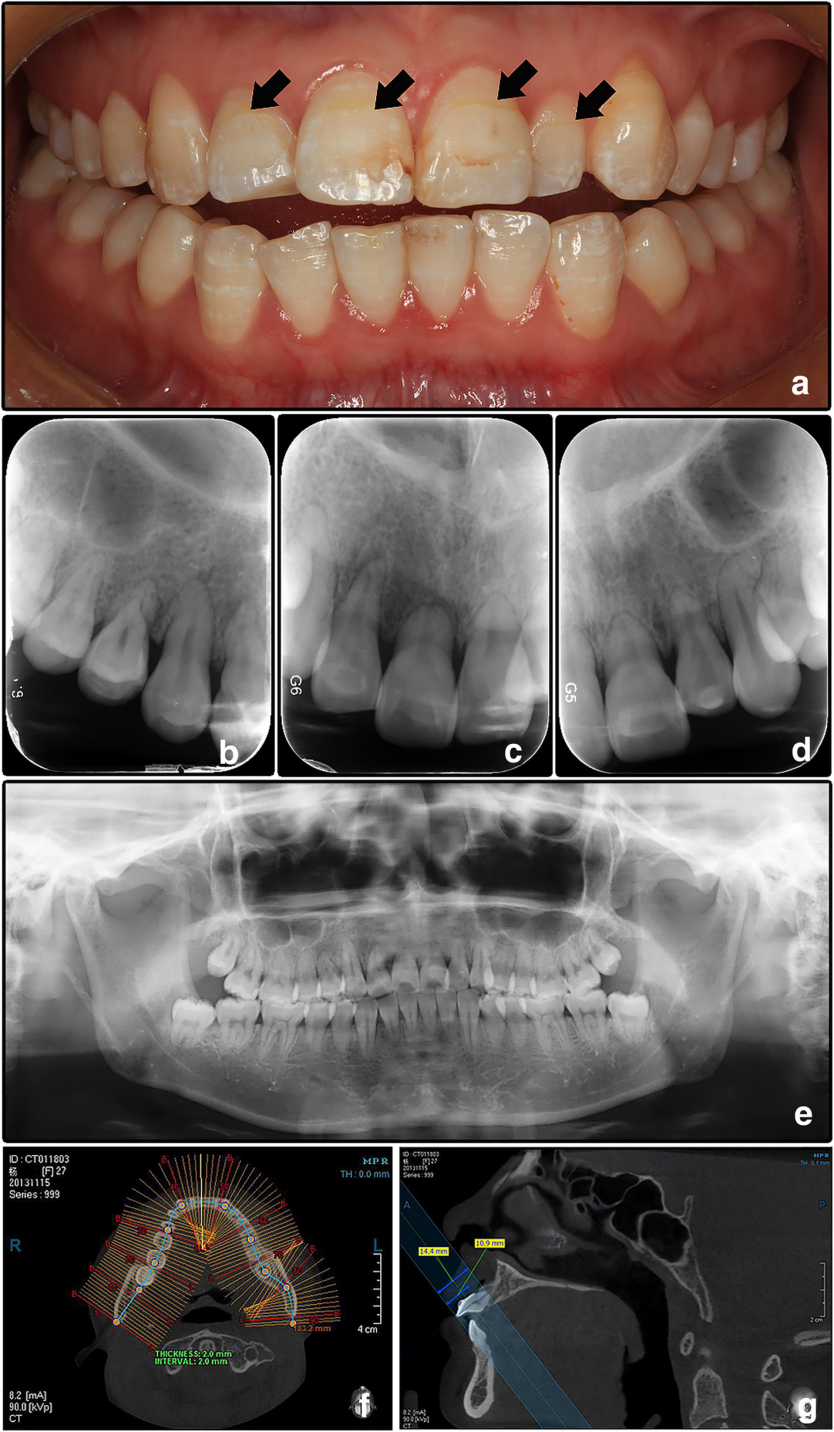
Clinical and imaging manifestation of Case 1. **a** Frontal intraoral view shows the teeth crowns appear normal except for several spots due to mild enamel hypoplasia. **b**-**d** Periapical radiographs show the short root anomaly of the maxillary incisors. **e** Panoramic film. **f**-**g** Teeth lengths were measured by CBCT

A 27-year-old female Chinese patient presented with looseness of her maxillary incisors (teeth #11 and #12) was treated with an arch bar from tooth #14 to tooth #22 for 5 months in outside institution. She then suffered from upper incisors pain and was referred to us. The patient was 160 cm height and 46.5 kg weight, with a Body Mass Index (BMI) of 18.2 (normal range 18.5–23.9). She also had mild symptoms of heat intolerance, anxiety, polyphrasia, tremor, palpitate, chest tightness, anhelation, and muscle aches. The patient had a history of premature loss of some deciduous teeth in childhood. The family history was unremarkable. Physical examination revealed mild goiter and exophthalmos. Intraoral examination found pale spots on the crowns due to mild enamel hypoplasia (Fig. 1a). No tender to percussion, and no tooth mobility after removal of the arch bar.

Periapical and panoramic radiographs showed that the roots of all teeth were short (Fig. 1b-e).CBCT scan (Fig. 1f, g) proved that the lengths of all the roots (except for four 3rd molars) were shorter than normal, whereas the crowns lengths were broadly within the normal range (Table 1). Chest X-ray and skeletal radiographs of upper and lower limbs didn’t show any significant changes. The laboratory analyses of urinalysis, complete blood count, serum calcium level, serum CT level, and PTH level were normal. Serum ALP level was 59.0 U/L (normal range 45.0–125.0 U/L). Unexpectedly, serum TSH level was lower than 0.011 uIU/mL (normal range 0.270–4.200 uIU/mL), serum FT3 7.52 pmol/L (normal range 3.10–6.80 pmol/L), and serum FT4 18.33 pmol/L (normal range 12.00–22.00 pmol/L).

**Table 1:** Crown length, root length and tooth length of patient one

	Crown length			Root length			Tooth length		
	CL (mm)	n CL (mm)	n CLR (mm)	RL (mm)	n RL (mm)	n RLR (mm)	TL (mm)	n TL (mm)	n TLR (mm)
#11	10.9	11.2	8.6–14.7	3.5 ^a^	13.0	6.3–20.3	14.4 ^a^	23.6	16.5–32.6
#21	10.6			4.0 ^a^			14.6 ^a^		
#12	8.2	9.8	7.4–11.9	7.0 ^a^	13.4	9.6–19.4	15.2 ^a^	22.5	17.7–28.9
#22	8.5			5.7 ^a^			14.2 ^a^		
#13	8.6	10.6	8.2–13.6	8.2 ^a^	16.5	10.8–28.5	16.8 ^a^	26.4	20.0–38.4
#23	8.2			8.2 ^a^			16.4 ^a^		
#14	6.1^a^	8.6	7.1–11.1	7.3 ^a^	13.4	8.3–19.0	13.4 ^a^	21.5	15.5–28.9
#24	5.6^a^			6.6 ^a^			12.2 ^a^		
#15	4.8^a^	7.7	5.2–10.5	7.8 ^a^	14.0	8.0–20.6	12.6 ^a^	21.2	15.2–28.4
#25	5.7			7.7 ^a^			13.4 ^a^		
#16	6.1^a^	7.5	6.3–9.6	7.7 ^a^	12.9	9.3–17.3	13.8 ^a^	20.1	17.0–27.4
#26	5.3^a^			8.2 ^a^			13.5 ^a^		
#17	5.8^a^	7.6	6.1–9.4	8.1 ^a^	12.9	9.3–17.8	13.9 ^a^	20.0	16.0–26.2
#27	5.4^a^			8.0 ^a^			13.4 ^a^		
#18	6.2	7.2	5.7–9.0	7.1	10.7	7.1–15.3	13.2 ^a^	17.5	14.0–22.5
#28	6.0			7.5			13.5 ^a^		
#41	6.6	8.8	6.3–11.6	7.1 ^a^	12.6	7.7–17.9	13.7 ^a^	20.8	16.9–26.7
#31	6.4			6.8 ^a^			13.2 ^a^		
#42	7.8	9.4	7.3–12.6	6.9 ^a^	13.5	9.4–18.1	14.7 ^a^	22.1	18.5–26.6
#32	7.3			7.9 ^a^			15.2 ^a^		
#43	8.5	11.0	6.8–16.4	9.3 ^a^	15.9	9.5–22.5	17.8	25.9	16.1–34.5
#33	8.4			9.3 ^a^			17.7		
#44	7.6	8.2	5.9–10.9	8.8 ^a^	14.7	9.7–20.2	16.4 ^a^	22.4	17.0–28.5
#34	8.1			8.1 ^a^			16.2 ^a^		
#45	6.7	8.8	6.7–10.2	8.4 ^a^	14.4	9.2–21.2	15.1 ^a^	22.1	16.8–28.1
#35	7.1			7.2 ^a^			14.3 ^a^		
#46	6.3	7.7	6.1–9.6	8.9 ^a^	13.5	9.3–18.9	15.2 ^a^	20.9	17.0–27.7
#36	6.6			8.7 ^a^			15.3 ^a^		
#47	6.9	7.7	6.1–9.8	7.2 ^a^	13.5	8.9–18.8	14.1 ^a^	20.6	15.0–25.5
#37	6.5			8.2 ^a^			14.7 ^a^		
#48	6.5	7.5	6.1–9.2	6.8	11.2	6.3–14.3	13.3 ^a^	18.2	14.8–22.0
#38	6.6			6.7			13.3 ^a^		

*CL* crown length, *n CL* normal average crown length, *n CLR* normal range of crown length, *RL* root length, *n RL* normal average root length, *n RLR* normal range of root length, *TL* tooth length, *n TL* normal average tooth length, *n TLR*, normal range of tooth length

^a^ indicates that the length is shorter than the lower limit of the normal range. All the normal data are obtained from Woelfel’s Dental Anatomy [31]

With consultation by an endocrinologist, the patient was diagnosed as HPP (odontohypophosphatasia form) and hyperthyroidism [4, 5]. Since serum ALP level in patients with hyperthyroidism would be higher than normal [4, 6, 7], we suspected that this might compensate the reduction of ALP level caused by HPP and thus led to the false-negative result of serum ALP level in this case.

### Case two

**Fig. 2:**
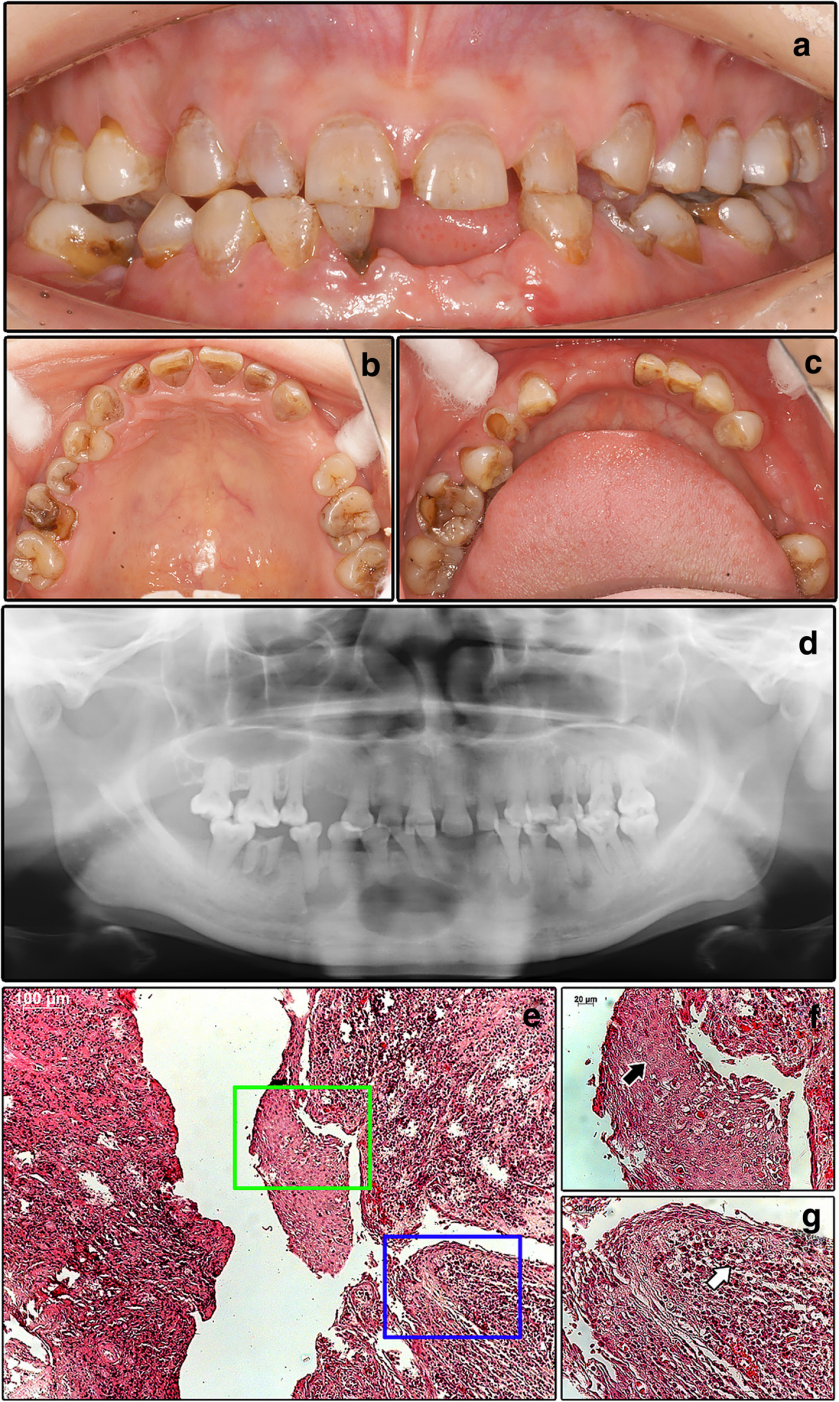
Clinical and imaging manifestation of Case 2. **a**-**c** Intraoral view shows the dysplasia. D. Panoramic film shows the short root anomaly. **e**-**g** Histological analysis of the radicular cysts. **e** cyst wall of the radicular cyst, with Rushton body (black arrow in **f**) and inflammatory cell infiltration (white arrow in **g**)

A 24-year-old female Chinese patient presented with repeated episodes of swelling and pain of her chin for 2 years. The patient had a history of premature loss of some deciduous teeth before 6-year-old and accidental leg fracture at 10-year-old. Family history revealed that her mother lost several teeth at 40-year-old. The patient was 160 cm height and 39 kg weight, with a BMI of 15.23. Intraoral examination (Fig. 2a-c) and CBCT showed enamel hypoplasia in most of her residual teeth. Teeth #14 and # 31 were missing. No fistula or pus was noted.

The panoramic film (Fig. 2d) revealed that all the roots were short, and multiple well defined radiolucencies were shown at the apices of teeth #25, #26, #32–35, and #41–46. Furthermore, alveolar bone height reduction was observed as well. CBCT proved that most of the root lengths were shorter than normal (Table 2). Skeletal radiography didn’t show any obvious changes. The laboratory examination results were unremarkable except that the serum ALP level was 35.0 U/L (normal range 45.0–125.0 U/L).

**Table 2:** Crown length, root length and tooth length of patient two

	Crown length			Root length			Tooth length		
	CL (mm)	n CL (mm)	n CLR (mm)	RL (mm)	n RL (mm)	n RLR (mm)	TL (mm)	n TL (mm)	n TLR (mm)
#11	6.3*	11.2	8.6–14.7	9.9	13.0	6.3–20.3	16.2*	23.6	16.5–32.6
#21	6.5*			9.8			16.3*		
#12	6.1*	9.8	7.4–11.9	8.9*	13.4	9.6–19.4	15.0*	22.5	17.7–28.9
#22	5.9*			9.3*			15.2*		
#13	6.1*	10.6	8.2–13.6	9.7*	16.5	10.8–28.5	15.8*	26.4	20.0–38.4
#23	6.2*			9.8*			16.0*		
#14	0.0*	8.6	7.1–11.1	0.0	13.4	8.3–19.0	0.0	21.5	15.5–28.9
#24	5.8*			9.0			14.8*		
#15	5.9	7.7	5.2–10.5	9.1	14.0	8.0–20.6	15.0*	21.2	15.2–28.4
#25	5.7			9.1			14.8*		
#16	6.1*	7.5	6.3–9.6	8.5*	12.9	9.3–17.3	14.6*	20.1	17.0–27.4
#26	5.8*			8.5*			14.3*		
#17	5.8*	7.6	6.1–9.4	8.9*	12.9	9.3–17.8	14.7*	20.0	16.0–26.2
#27	5.4*			8.8*			14.2*		
#18	0.0	7.2	5.7–9.0	0.0	10.7	7.1–15.3	0.0	17.5	14.0–22.5
#28	0.0			0.0			0.0		
#41	3.4*	8.8	6.3–11.6	9.4	12.6	7.7–17.9	12.8*	20.8	16.9–26.7
#31	0.0			0.0			0.0		
#42	4.4*	9.4	7.3–12.6	8.1*	13.5	9.4–18.1	12.5*	22.1	18.5–26.6
#32	3.1*			8.0*			11.1*		
#43	5.4*	11.0	6.8–16.4	10.1	15.9	9.5–22.5	15.5*	25.9	16.1–34.5
#33	5.2*			9.5			14.7*		
#44	5.0*	8.2	5.9–10.9	9.4*	14.7	9.7–20.2	14.4*	22.4	17.0–28.5
#34	4.9*			9.6*			14.5*		
#45	5.2*	8.8	6.7–10.2	10.0	14.4	9.2–21.2	15.2*	22.1	16.8–28.1
#35	5.1*			9.8			14.9*		
#46	1.4*	7.7	6.1–9.6	8.7*	13.5	9.3–18.9	10.1*	20.9	17.0–27.7
#36	5.8*			8.2*			14.0*		
#47	5.9*	7.7	6.1–9.8	8.5*	13.5	8.9–18.8	14.4*	20.6	15.0–25.5
#37	5.8*			8.1*			13.9*		
#48	0.0	7.5	6.1–9.2	0.0	11.2	6.3–14.3	0.0	18.2	14.8–22.0
#38	0.0			0.0			0.0		

Length 0.0 means the tooth was already lost

*indicates that the length is shorter than the lower limit of the normal range. All the normal data are obtained from Woelfel’s Dental Anatomy [31]

The patient was diagnosed as HPP (odontohypophosphatasia form) and multiple radicular cysts [5]. Root canal therapy of teeth #25, #26, #33–35, and #41–44 was performed before enucleation of the radicular cysts. In addition, teeth #32, #45, and #46 were extracted. Pathological report confirmed the diagnosis of radicular cysts (Fig. 2e-g).

## Discussion

In the present study, two cases of odontohypophosphatasia form of HPP were reported, one concurrent with hyperthyroidism, and the other concurrent with multiple radicular cysts. To our knowledge, this is the first report that multiple radicular cysts and hyperthyroidism presented with odontohypophosphatasia form of HPP.

HPP patients possess mutations in the TNSALP gene, resulting in low serum level or activity of ALP, which can cause mineralization disorders and finally progress to bone or tooth dysplasia [5]. More than 270 distinct mutations and 15 polymorphisms have been recorded so far [8]. Different forms of HPP have quite different clinical manifestations despite the similar pathogenesis. Perinatal (lethal) form is an autosomal recessive disease and mostly the fetus would die of dyspnoea or the dysfunction of bone formation [9]. Prenatal (benign) form is an autosomal recessive or autosomal dominant disease presenting with obvious long bone deformity in infantile period without serious bone mineralization defects. Infantile form is an autosomal recessive disease and also generally considered lethal since the infants would suffer from dyspnoea caused by respiratory complication. Childhood form is an autosomal recessive or autosomal dominant disease presenting with several oral and systemic symptoms in children [10]. Adult form is an autosomal recessive or autosomal dominant disease which presents with recurrent stress fractures, thigh pain, osteoarthropathy, and premature loss of deciduous and/or permanent teeth [11]. Odontohypophosphatasia form is an autosomal recessive or autosomal dominant disease, which seems to present only in a dental phenotype. The diagnosis of this form remains controversial. It is characterized by premature loss of deciduous and/or permanent teeth (commonly the incisors), but not associated with deformities of the bone. The affected teeth may present dentin or cemental hypoplasia, enlarged root canals and pulp chambers. Biochemical findings are generally not considered as gold standard because the serum ALP level could be only slightly lower than normal, or even within the normal range [8, 12, 13]. Therefore any patient with a history of unexplainable premature loss of deciduous and/or permanent teeth should be suspected [13].

Although conspicuously different from each other in clinical manifestations, the oral symptoms are quite similar among varied forms. Premature loss of deciduous and/or permanent teeth is significant for diagnosis, which presented in both patients in our study. Alveolar bone height reduction, short root anomaly, and expanding root or pulp chamber are common oral manifestations [14–17]. Additionally, enamel hypoplasia, dentin dysplasia and cemental hypoplasia are also frequently observed [18]. Periodontal diseases such as gingivitis and periodontitis are rare in HPP [19]. Our present study firstly reported that multiple radicular cysts occurred in patient with odontohypophosphatasia form of HPP. It is believed that cemental hypoplasia, especially acellular cemental hypoplasia, plays a major part in the oral manifestation [20]. Inorganic pyrophosphate (PPi) acts as an indispensable regulator for root acellular cementum development, and is a key determinant that defines the hard-soft interface between the periodontal ligament and cementum. Acellular extrinsic fiber cementum, a tissue vital to tooth attachment and function, would be affected by high PPi level resulting from low ALP activity. That is to say, low serum ALP level or activity could lead to severe dysplasia or even absence of acellular cementum. Acellular cemental hypoplasia could influence the development of root and periodontal ligament attachment which would eventually result in premature loss of deciduous and/or permanent teeth [20].

A definitive diagnosis of HPP is usually not so distinct to be made. Although skeletal radiographs are easy to obtain, patients with odontohypophosphatasia form of HPP may not present any bone deformities such as craniomalacia or rachitic changes [5]. With the use of CBCT, minor structural changes of teeth would be identified. Serum ALP level or activity, which could be detected easily, indicates the severity of HPP, but can sometimes show false-negative [4, 21, 22]. Diseases such as hepatic carcinoma, hyperthyroidism and multiple radicular cysts may present high serum ALP level and therefore interfere the result [4, 21, 22]. The acellular cementum is more sensitive than bone tissue to serum ALP level change, since PPi plays an important role in acellular cementum development [20]. Genetic analysis can detect the mutation genes for the diagnosis of this inherited disease [23]. In addition, the differential diagnosis of X-linked hypophosphatemia, caused by the mutation in phosphate-regulating gene with homologies to endopeptidases on the X chromosome (PHEX) gene sequence, should be taken into consideration. With reduced PHEX enzymatic activity, osteopontin could accumulate in the bone and cause osteomalacia [24]. In our cases, symptoms of hypophosphatemia and osteomalacia were not observed.

It is generally consented that, the earlier the onset of HPP is, the more severe the disease gets, and the worse the prognosis will be. Although some symptoms could relieve spontaneously in mild form, the management is still under investigation [14]. High phosphate preparations like neutral sodium phosphate or biphosphonates could elevate serum phosphate level and increase the urinary excretion of PPi [25]. Surgical treatment could alleviate bone deformities [26]. Bone marrow transplantation can mitigate the respiratory complication and bone deformities in perinatal form patients [27]. Enzyme replacement therapy, high-dose subcutaneous injection of a mineral-targeting human TNSALP (sALP-FcD10), could prevent the symptoms of infantile form HPP in TNSALP-null mice [28]. What’s more, the enzyme replacement therapy asfotase alfa (Strensiq™; Alexion Pharmaceuticals Inc, New Haven, CT, USA), a recombinant human TNSALP, showed remarkable curative effect such as improvements in skeletal manifestation, pulmonary function, and growth in perinatal or infantile form HPP patients [29]. Recently asfotase alfa was approved in the USA, Canada, and Europe for the treatment of perinatal/infantile- and juvenile-onset HPP, and in Japan for the treatment of HPP [18]. Another exciting field is the potential application of genetic therapy. Injection of the viral vector with TNSALP gene to mice could maintain the serum TNSALP at a high level and alleviate the symptoms, which also provides a promising approach in the management of HPP in the future [30]. In the present study, the first patient achieved relief of symptoms after removal of arch bar and kept asymptomatic during 24 months follow-up. The second case was treated with enucleation of radicular cysts after the root canal treatment of the affected teeth. The patient was followed up for 21 months without any symptoms.

## Conclusions

Odontohypophosphatasia form of hypophosphatasia is a rare disease which is difficult to be diagnosed because of lack of systemic symptoms. This report presents two cases of odontohypophosphatasia concurrent with hyperthyroidism or multiple radicular cysts, and highlights that the history of premature loss of deciduous and/or permanent teeth, oral manifestation and lab results provide critical information for clinical diagnosis.

## Abbreviations

ALP, alkaline phosphatase; BMI, body mass index; CBCT, cone-beam computed tomography; CT, calcitonin; FT3, free triiodothyronine; FT4, free thyroxine; HPP, hypophosphatasia; TNSALP, tissue non-specific alkaline phosphatase; PTH, parathyroid hormone; TSH, thyroid-stimulating hormone; PHEX, phosphate-regulating gene with homologies to endopeptidases on the X chromosome (also known as Phosphate-regulating neutral endopeptidase, X-linked)

## References

[1] Whyte MP (1994). Hypophosphatasia and the role of alkaline phosphatase in skeletal mineralization. Endocr Rev.

[2] Fraser D (1957). Hypophosphatasia. Am J Med.

[3] Kim S (2012). Endodontic application of cone-beam computed tomography in South Korea. J Endod.

[4] Clark VL, Kruse JA (1990). Clinical methods: The history, physical, and laboratory examinations. JAMA.

[5] Reibel A, Maniere MC, Clauss F, Droz D, Alembik Y, Mornet E (2009). Orodental phenotype and genotype findings in all subtypes of hypophosphatasia. Orphanet J Rare Dis.

[6] Akalin A, Colak O, Alatas O, Efe B (2002). Bone remodelling markers and serum cytokines in patients with hyperthyroidism. Clin Endocrinol (Oxf).

[7] Isaia GC, Roggia C, Gola D, Stefano MD, Gallone G, Aimo G (2000). at al. Bone turnover in hyperthyroidism before and after thyrostatic management. J Endocrinol Invest.

[8] Whyte MP (2010). Physiological role of alkaline phosphatase explored in hypophosphatasia. Ann N Y Acad Sci.

[9] Tadokoro M, Kanai R, Taketani T, Uchio Y, Yamaguchi S, Ohgushi H (2009). New bone formation by allogeneic mesenchymal stem cell transplantation in a patient with perinatal hypophosphatasia. J Pediatr.

[10] Simon-Bouy B, Taillandier A, Fauvert D, Brun-Heath I, Serre JL, Armengod CG (2008). Hypophosphatasia: molecular testing of 19 prenatal cases and discussion about genetic counseling. Prenatal Diag.

[11] Whyte MP, Essmyer K, Geimer M, Mumm S (2006). Homozygosity for TNSALP mutation 1348c > T (Arg433Cys) causes infantile hypophosphatasia manifesting transient disease correction and variably lethal outcome in a kindred of black ancestry. J Pediatr.

[12] Mornet E (2008). Hypophosphatasia. Best Pract Res Clin Rheumatol.

[13] Collmann H, Mornet E, Gattenlohner S, Beck C, Girschick H (2009). Neurosurgical aspects of childhood hypophosphatasia. Childs Nerv Syst.

[14] Hollis A, Arundel P, High A, Gray JJ, Lemire I, Heft R (2013). Current concepts in hypophosphatasia: case report and literature review. Int J Paediatr Dent.

[15] Foster BL, Sheen CR, Hatch NE, Liu J, Cory E, Narisawa S (2015). Periodontal Defects in the A116T Knock-in Murine Model of Odontohypophosphatasia. J Dent Res.

[16] Wei KW, Xuan K, Liu YL, Fang J, Ji K, Wang X (2010). Clinical, pathological and genetic evaluations of Chinese patients with autosomal-dominant hypophosphatasia. Arch Oral Biol.

[17] Foster BL, Nagatomo KJ, Tso HW, Tran AB, Nociti FH, Narisawa S (2013). Tooth root dentin mineralization defects in a mouse model of hypophosphatasia. J Bone Miner Res.

[18] Bloch-Zupan A (2016). Hypophosphatasia: diagnosis and clinical signs - a dental surgeon perspective. Int J Paediatr Dent.

[19] Bloch-Zupan A, Alembik Y, Doray B, Linglart A, Mornet E, Morrier JJ (2012). Oro-dental features in hypophosphatasia: a valuable phenotype for disease diagnosis and evaluation of future treatment outcomes. Bull Group Int Rech Sci Stomatol Odontol.

[20] Foster BL, Nagatomo KJ, Nociti FH, Fong H, Dunn D, Tran AB (2012). Central role of pyrophosphate in acellular cementum formation. PLoS One.

[21] Zecchi-Orlandini S, Formigli L, Giannelli M, Martini M, Tonelli P, Brandi ML (1996). Radicular cysts are involved in the recruitment of osteoclast precursors. J Oral Pathol Med.

[22] Xu XS, Wan Y, Song SD, Chen W, Miao RC, Zhou YY (2014). Model based on γ-glutamyltransferase and alkaline phosphatase for hepatocellular carcinoma prognosis. World J Gastroenterol.

[23] Mornet E, Hofmann C, Bloch-Zupan A, Girschick H, Le Merrer M. Clinical utility gene card for: hypophosphatasia - update 2013. Eur J Hum Genet. 2014;22(4). doi:10.1038/ejhg.2013.177. 10.1038/ejhg.2013.177 PMC3953904 23921539

[24] Barros NM, Hoac B, Neves RL, Addison WN, Assis DM, Murshed M (2013). Proteolytic processing of osteopontin by PHEX and accumulation of osteopontin fragments in Hyp mouse bone, the murine model of X-linked hypophosphatemia. J Bone Miner Res.

[25] Mimoun E, Moulin P, Sales De Gauzy J, Mornet E, Salles JP (2012). Biphosphonates in hypophosphatasia: not the evil?. Bull Group Int Sci Somatol Odontol.

[26] Cole DE (2008). Hypophosphatasia update: recent advances in diagnosis and treatment. Clin Genet.

[27] Cahill RA, Wenkert D, Perlman SA, Steele A, Coburn SP, McAlister WH (2007). Infantile hypophosphatasia: Transplantation therapy trial using bone fragments and cultured osteoblasts. J Clin Endocr Metab.

[28] McKee MD, Nakano Y, Masica DL, Gray JJ, Lemire I, Heft R (2011). Enzyme replacement therapy prevents dental defects in a model of hypophosphatasia. J Dent Res.

[29] Whyte MP, Greenberg CR, Salman NJ, Bober MB, McAlister WH, Wenkert D (2012). Enzyme-replacement therapy in life-threatening hypophosphatasia. N Engl J Med.

[30] Yamamoto S, Orimo H, Matsumoto T, Iijima O, Narisawa S, Maeda T (2011). Prolonged survival and phenotypic correction of Akp2(−/−) hypophosphatasia mice by lentiviral gene therapy. J Bone Miner Res.

[31] Scheid R, Woelfel J (2007). Woelfel’s Dental Anatomy: Its Relevance to Dentistry.

